# From individual resilience to collective response: reframing ecological emotions as catalysts for holistic environmental engagement

**DOI:** 10.3389/fpsyg.2024.1363418

**Published:** 2024-06-06

**Authors:** Shicun Qiu, Jiacun Qiu

**Affiliations:** ^1^School of Foreign Languages, Sichuan University of Arts and Science, Dazhou, Sichuan, China; ^2^Business School, Guangdong Business and Technology University, Zhaoqing, Guangdong, China

**Keywords:** ecological emotions, intervention strategies, individual resilience, collective engagement, unity of humans and nature

## Abstract

The ongoing international study on the mental health implications of climate change has prompted a deeper exploration of ecological emotions such as eco-anxiety, eco-worry and eco-grief, which are associated with environmental degradation and the escalating climate crisis. Although psychological and mental health literature has mainly presented preliminary conceptual analyses, the understanding of ecological emotions remains unclear. This narrative review aims to clarify the definition, highlight precipitating factors, and outline the effects of ecological emotions on mental health, emphasizing the need for thorough research to shift the nonclinical intervention approach from merely promoting individual resilience to encouraging collective engagement. Our analysis of the literature reveals that the existing theoretical framework, which predominantly focuses on bolstering individual resilience, provides only temporary relief for acute symptoms without addressing the foundational social and environmental factors that trigger these ecological emotions. We conclude that it is crucial to overcome the limitations of Western anthropocentrism’s human-to-human interaction approach and embrace the unity of humans and nature to effectively manage the increasing ecological emotions. This perspective draws insights from the holistic and collective wisdom of indigenous cultures and traditional Chinese philosophy, offering a potential pathway toward maintaining a sustainable emotional balance amid the worsening global ecological turmoil.

## Introduction

Ecological disasters like climate change are increasingly recognized as one of the most severe threats to human health in the 21st century. The Intergovernmental Panel on Climate Change (IPCC) (2022) suggested in February 2022 that climate change would result in numerous risks to natural ecosystems and human health worldwide from 2040 onwards. The World Health Organization (2021) predicts that climate change will lead to 250,000 deaths annually between 2030 and 2050 due to malnutrition, malaria, diarrhea and heat stress. There is a growing body of evidence linking climate change, the likelihood of extreme weather events, and their impacts on health. As the world grapples with complex and often deteriorating global environmental issues, it becomes crucial to understand people’s reactions and emotional responses to these problems.

Since global awareness of ecological crises is growing rapidly and there is widespread media coverage of this issue, people who are aware of current and future threats posed by ecological issues may experience fear and anxiety about potential negative outcomes for their future and the world, particularly among those vulnerable populations. Yet, in recent debates surrounding the psychological impacts of ecological crises, there has been a tendency to pathologize ecological emotions—anxiety, worry, grief, and despair experienced in response to environmental degradation—as merely symptoms of disorder within individuals. For instance, surveys conducted across various countries revealed that many individuals experience high levels of eco-anxiety and eco-worry (Steentjes et al., 2017; Ballew et al., 2018; Minor et al., 2019; Gregersen et al., 2020). However, this narrow, individualistic interpretation fails to acknowledge the potential of these emotions to foster a collective consciousness and a unified response to the pressing challenges of climate change. In this narrative review, we propose a paradigmatic shift, framing eco-emotions not as individual pathologies but rather as natural responses to the recognition of our interdependence with the Earth. By cultivating an understanding of eco-emotions as motivators for community action and engagement, we aim to transcend the limitations of the traditional psychological perspective, opening the door to a more holistic approach that integrates human emotions, social dynamics, and the environment in the pursuit of a sustainable future.

## Aim and search strategy

Despite the prevalence of ecological emotions engendered by knowledge and experiences of the ecological crisis, the exact meaning of ecological emotions like eco-anxiety remains unclear and inconsistent. This narrative review seeks to summarize and discuss previous studies about the role ecological emotions play in people’s lives. By analyzing and synthesizing literature on key ecological emotions such as eco-anxiety, eco-anger, eco-worry, eco-sadness, ecological grief, eco-paralysis and eco-nostalgia, we aim to enhance our understanding of how climate change and other environmental issues impact human emotions and actions. The primary goal of this study is to conduct a narrative review of the literature on the range of emotional responses to the ecological crisis, thereby discerning the definition, causes and effects of ecological emotions. Delving into the dual nature of these emotions, this narrative review examines their correlation with mental health and their potential to foster constructive engagement on both an individual and a collective level. Furthermore, this review underscores the need for high-quality research to explore the potential shift in nonclinical intervention strategies from fostering individual resilience to promoting collective engagement.

It is pertinent to note that, as a narrative rather than a systematic review, the primary objective here is not an exhaustive compilation of extant literature on the emotional dimensions of environmental issues. Accordingly, this narrative review was conducted by comprehensively searching electronic databases including PubMed, PsycINFO, Scopus, Web of Science and CNKI and the keywords used in the search were “ecological emotions,” “eco-anxiety,” “eco-anger,” “eco-worry,” “eco-sadness,” “ecological grief,” “eco-paralysis,” and “eco-nostalgia.” These terms were used in various combinations with AND/OR operators to ensure comprehensive retrieval of relevant literature. Studies included in this review were peer-reviewed articles published in English from January 1980 to November 2023. The selection focused on articles that presented theoretical analyses, qualitative data, and narrative reviews related to ecological emotions and their psychological impacts. Exclusion criteria ruled out non-peer-reviewed articles, conference abstracts, and studies not specifically addressing ecological emotions as defined by our keywords. Studies merely focusing on general environmental concerns without addressing specific emotional responses were also excluded. At the same time, we aimed to include a variety of perspectives to capture a wide range of experiences and ideas. This involved considering works from different geographical regions and cultural backgrounds to ensure a comprehensive understanding of the global impact of ecological emotions.

## Definitions of focal ecological emotions

In the contemporary discourse, the term *ecological* predominantly references the natural environment; however, its semantic scope extends further to encompass a sense of community and the socio-political atmosphere, signifying the collective effect engendered within and by a group of individuals (Kałwak and Weihgold, 2022). From a biological perspective, *emotion* is posited as the paramount sensory mechanism through which humans discern their relationship with the surrounding world, rendering it indispensable for the survival and social cohesion of human collectives (Hochschild, 2012). Hochschild emphasizes the significance of collective life, a theme that resonates at the heart of the nature and genesis of ecological emotions. This dual interpretation of *ecological*, encompassing both the environmental and the social realms, proves critical to this narrative review, highlighting the intricate interplay between these dimensions. Consequently, the concept of ecological emotions can be defined as a sensory capacity that critically informs individuals about the evolving dynamics within their natural and social environment (Kałwak and Weihgold, 2022).

To be more specific, ecological emotions at least include eco-anxiety, eco-anger, eco-worry, eco-sadness, ecological grief, eco-paralysis and eco-nostalgia. To begin with, the concept of eco-anxiety resides within the broader domain of anxiety, referring to negative emotions like worry, unease, tension, and fear that individuals display in response to stressful events or crises (Spielberger et al., 1983). Trait anxiety and state anxiety are two key categories for understanding anxiety. Trait anxiety tends to obstruct normal life functioning and constitutes a significant threat to mental and physical health (Myles et al., 2020), clinching its status as a mental and emotional disorder in clinical psychology (Spalding et al., 2021). In contrast, as an immediate individual response to external stimuli, state anxiety is non-clinical and ubiquitous (Weeks et al., 2019), inducing individuals to engage in various coping strategies to alleviate and eliminate the discomfort it brings (Gino et al., 2012).

Just like anxiety, eco-anxiety is often understood from both clinical and non-clinical perspectives. As one of the pioneers of the study of psychoneurotic syndromes, Albrecht (2011) argued that mental health effects result from negative emotions triggered by perceived environmental crises like climate change, and as one of these negative emotions, eco-anxiety could be categorized as people’s reactions of worry and anxiety concerning global climate change threats and concurrent environmental degradation. Clayton and Karazsia (2020) designed a measure of climate change anxiety that includes not only pure emotional aspects such as worry and anxiety but also rumination and functional impairment. Dodds (2021) also delineated eco-anxiety as a chronic mental health condition emanating from ecological hazards, paralleling the characteristics of trait anxiety. Seen from this clinical perspective, at least in three studies (Schwartz et al., 2022; Patrick et al., 2023; Reyes et al., 2023), eco-anxiety was gaged as functional impairment (interference of concern about climate change with an individual’s capacity to work or socialize) and as cognitive-emotional impairment (such as rumination, difficulty sleeping or concentrating, and nightmares or crying due to climate change). When eco-anxiety becomes unmanageable and begins to interfere with an individual’s daily functioning, it is perceived as clinically meaningful (Clayton, 2020). Based on a non-clinical perspective, however, eco-anxiety can spur environmentally sustainable behaviors (Pihkala, 2020a), thereby not necessarily indicative of a clinical diagnosis. As individuals might experience short-term state anxiety responses based on external environmental stimuli and negative information, some researchers have started recognizing eco-anxiety not just as a negative psychosocial impact resulting from environmental challenges but also as individuals’ constructively adaptive and state anxiety response toward ecological threats (Wullenkord et al., 2021; Whitmarsh et al., 2022).

Specifically, most researchers have assessed eco-anxiety as an array of negative emotions in response to climate change, including feelings of anxiety (Searle and Gow, 2010; Hickman et al., 2021; Stanley et al., 2021), worry (Searle and Gow, 2010; Berry and Peel, 2015; Hickman et al., 2021; Sciberras and Fernando, 2021), tension (Searle and Gow, 2010), helplessness, powerlessness, sadness, depression, anger (Searle and Gow, 2010; Hickman et al., 2021), grief, guilt, (Hickman et al., 2021), and fear (Hickman et al., 2021; Stanley et al., 2021). In this way, eco-anxiety is considered a specific emotional response to ecological problems. As to the essential characteristics of eco-anxiety, they include an individual’s threat assessment of climate changes and other environmental dilemmas as well as an inherent uncertainty, with the latter implying that the sources of eco-anxiety lie in future environmental uncertainties (Clayton, 2020). Up to now, however, there is no standard definition of eco-anxiety. Consequently, diverse definitions are deployed, including an enduring fear of environmental catastrophe (Clayton et al., 2017), having habitual worrying thoughts (Verplanken et al., 2020), anxiety stemming from current and forecasted environmental damage or loss (Ojala et al., 2021), functional or cognitive-emotional impairments (Schwartz et al., 2022; Reyes et al., 2023), and a constant, difficult-to-control apprehensiveness and worry about climate change (van Valkengoed et al., 2023).

Beyond eco-anxiety, environmental challenges may induce other negative emotions like anger, worry, sadness, and grief. Although these emotions may emanate from the same environmental issues, they usually have unique connotations. For instance, eco-anger arises from others’ destructive behavior or governmental agencies’ inaction (Myers et al., 2012). While eco-anxiety was found to correlate with a decrease in collective action or withdrawal from pro-climate initiatives, eco-anger was linked to increased participation in collective action (Stanley et al., 2021). Eco-worry envelops worry for the present and predicted ecological damage and destruction (Ojala et al., 2021). Eco-sadness signifies sadness stemming from experienced or anticipated environmental losses of ecosystems and species, a sensation particularly strong in groups with tight bonds to nature (Cunsolo and Ellis, 2018). Eco-grief refers to sorrow and grief experienced in response to the loss of cherished locations, ecosystems, and species (Ojala et al., 2021). As a reaction to environmental degradation and loss of species and beloved environments, eco-grief incites various emotional responses, such as frustration, fear, stress, distress, hopelessness, and pre- and post-traumatic stress disorder, which lead to a sense of lost identity (Cunsolo and Ellis, 2018; Marshall et al., 2019; Cunsolo et al., 2020a,b). Often classified as a form of disenfranchised grief, eco-grief is often publicly unacknowledged or downplayed through socio-economic and socio-cultural structures and policies (Adger et al., 2017; Cunsolo and Ellis, 2018).

This section begins with an overview of ecological emotions, drawing from a broad array of foundational texts to set the stage for a deeper exploration of specific emotional responses and their triggers in the next sections.

## Causes of ecological emotions

While the study of ecological emotions is relatively nascent, initial investigations have identified several internal and external factors as potential catalysts. Internally, demographic characteristics and personal attributes appear impactful. Externally, our emotions are shaped by direct or indirect experiences with climate change and other environmental problems.

### Internal factors

Firstly, demographic characteristics heavily influence the discourse on ecological emotions. For instance, while women may be more prone to eco-anxiety due to increased perceived pressure, one study argued that gender had an insignificant impact on such emotion when subjective experiences were accounted for (Chen et al., 2020). Concerning age, a study suggested that, due to their health conditions and limited mobility, older adults might be more susceptible to eco-anxiety (Chen et al., 2020). Other studies argued that younger individuals might experience increased eco-anxiety, as they better understood climate change’s implications and held greater concerns about its future impacts (Clayton, 2020; Dodds, 2021).

The existing body of research has also examined the impact of individual traits on ecological emotions. Individuals displaying a higher level of concern toward the environment are more likely to perceive changes in the ecological environment, and thereby more susceptible to eco-anxiety (Searle and Gow, 2010). Similarly, mental health conditions such as trait anxiety and depression could potentially heighten individual vulnerability to ecological emotions (Wullenkord et al., 2021). Personality traits related to emotional processing and regulation might also contribute to ecological emotions, making neurotic individuals more susceptible to negative reactions (Chen et al., 2020). Furthermore, individuals of lower socioeconomic status, owing to limited economic and health resources, might display higher levels of ecological emotions (Dodds, 2021).

Lastly, acting as influential factors of ecological emotions, cognitive and motivational factors generally further activate emotional responses. An essential condition for the emergence of ecological emotions is that environmental problems constitute a threat substantial enough to become a source of pressure for an individual. Therefore, ecological emotions are often associated with perceptions of risk and threats (Chen et al., 2020), and individuals who deny environmental problems are very likely to report lower levels of ecological emotions (Wullenkord et al., 2021). Moreover, ecological emotions positively correlate with obstructed needs and inversely with satisfied ones (Wullenkord et al., 2021).

### External factors

Ecological emotions, fundamentally reactions to external environmental stimuli, are tied to direct or indirect experiences of climate change and similar issues (Hogg et al., 2021). Direct experience refers to the situations where an individual directly experiences environmental problems as well as their negative impacts. Typically, individuals who have experienced or have been affected by some environmental problems are more susceptible to ecological emotions (Clayton, 2020). For instance, those strongly attached to their environment might feel heightened eco-anxiety when it undergoes significant alterations (Chen et al., 2020) and those individuals living in areas more susceptible to environmental impacts might also have higher levels of eco-anxiety (Clayton, 2020). Just as some researchers argued, experiences of flooding were linked to escalated concern and heightened psychological importance of climate change, subsequently predicting behavioral intentions and support for climate change policy (Spence et al., 2011; Demski et al., 2017). Negative emotions tied to climate change have also been documented among those enduring prolonged droughts in Australia (Ellis and Albrecht, 2017).

Besides direct experience, exposure to climate change data through the media also plays a vital role in the public’s risk perception and evaluation (Clayton, 2020; Budziszewska and Jonsson, 2021). To a great extent, how the public receives information on environmental problems determines the level of eco-anxiety (Ojala et al., 2021). Ogunbode et al. (2020) once demonstrated that the IPCC special report on 1.5°C global warming significantly amplified climate change concerns among 75% of Norwegians who had learned about the report via traditional television and print media. Undeniably, with rising public concern, media coverage of climate change has escalated globally (Sampei and Aoyagi-Usui, 2009; Carmichael and Brulle, 2018). Moreover, personal values and worldviews influence responsiveness to media content, and individuals tend to interpret media reports about ecological issues based on their frames of reference (Höijer, 2004; Newman et al., 2018).

Finally, political ideology and cultural beliefs play an important role in whether people subjectively attribute ecological emotions to environmental crises. Conservative individuals who typically express scepticism toward anthropogenic climate change might not necessarily feel more anxious despite experiencing severe weather (Whitmarsh, 2011). Hickman et al. (2021) suggested that perceptions of governmental inaction or inadequate response might lead to higher levels of eco-anxiety which is more likely to provoke unconstructive defense mechanisms. The environmental values such as the new ecological paradigm and connection with nature can also exert influence over ecological emotions (Whitmarsh et al., 2022). Emphasizing mutual reliance and respect for nature, indigenous people are more susceptible to eco-anxiety in the face of environmental crises (Clayton, 2020).

## Effects of ecological emotions on mental health

Currently, ecological emotions, especially eco-anxiety, are primarily considered clinical mental disorders or chronic psychological diseases triggered by environmental crises, precipitating a focus on the deleterious effects of ecological emotions on individuals’ mental health.

Numerous studies have found that heightened ecological emotions are associated with various mental health problems. Symptoms of depression (Searle and Gow, 2010; Sciberras and Fernando, 2021; Schwartz et al., 2022), anxiety (Searle and Gow, 2010; Stanley et al., 2021; Schwartz et al., 2022), stress (Searle and Gow, 2010; Stanley et al., 2021), psychological distress (Reyes et al., 2023), and post-traumatic stress disorder (PTSD) (Patrick et al., 2023) have all been linked with higher level of ecological emotions.

To be specific, some studies treated eco-anxiety as a clinically pathological emotion negatively affecting mental health and physiological responses. Swim et al. (2009) argued that eco-anxiety could induce clinical physiological symptoms such as panic attacks, loss of appetite, irritability, physical weakness and insomnia. Moreover, clinical eco-anxiety could provoke other negative emotional responses like depression, despair and powerlessness (Weintrobe, 2012). Furthermore, anxiety stemming from the awareness of global climate threats might exacerbate clinical levels of depressive and anxious symptoms, meaning that eco-anxiety might negatively impact mental health (Searle and Gow, 2010). In fact, chronic distress from ecological emotions might make it hard to control negative emotions and potentially interfere with individuals’ sleep, social skills and work abilities (Clayton, 2020). Clayton and Karazsia (2020) argued that eco-anxiety has damaging characteristics such as cognitive-emotional damage like difficulty sleeping and crying without reason, and functional damage such as difficulty engaging in entertainment and decreased potential work abilities. When summarizing cognitive damage caused by eco-anxiety, Hogg et al. (2021) also mentioned that chronic eco-anxiety might cause obsessive-compulsive disorder. At last, after examining the relationship between eco-anxiety and subjective well-being and mental health among Gen Z youth in the Philippines, Reyes et al. (2023) concluded that eco-anxiety negatively affects mental health.

In addition, due to worries about future environmental conditions, ecological emotions might also make people question the choice of having children (Clayton, 2020). In a survey composed of climate-concerned participants, all commented that not having children was their greatest contribution to the environment (Helm et al., 2021). To a great extent, having fewer or no children because of climate-related concerns can be seen as a manifestation of ecological emotions: participants expressed serious concern, anxiety, and even anguish about the climate impacts that children would have to face during their lives (Schneider-Mayerson and Leong, 2020; Helm et al., 2021).

As a result, ecological emotions evoked by the impending menace of climate change can be either adaptive or maladaptive (Taylor, 2020). While adaptive eco-emotions can spark climate activism including initiatives to reduce carbon footprint, maladaptive eco-emotions may culminate in anxious passivity, an inability to confront ecological crises. This could further take the form of an anxiety disorder triggered or amplified by climatic stressors. Comparatively speaking, experiencing eco-anger was found to predict improved mental health outcomes as well as heightened participation in pro-climate activism and personal behaviors. On the contrary, eco-anxiety and eco-worry were less adaptive, correlating with diminished well-being. These findings emphasized anger as a crucial adaptive emotional driver of engagement with ecological crises (Stanley et al., 2021).

## Negative impacts of ecological emotions on vulnerable populations

This section will integrate studies focusing on how ecological crises disproportionately affect vulnerable groups, highlighting the intensity and range of emotional responses triggered by such disparities.

It is worth emphasizing that the negative impacts of ecological crises do not affect all global populations equally (Ojala et al., 2021). With limited resources to utilize, those most likely to experience these impacts include people residing in eco-sensitive communities and regions reliant on natural resources, numerous indigenous natives, and individuals with pre-existing health conditions or stressors (Cunsolo and Ellis, 2018; Tschakert et al., 2019). Although such vulnerable groups historically have been underrepresented in global climate change discourse, recent research has started to focus more on these demographics and their unique mental health challenges. Studies have delved into the psychological effects of long-term drought and solastalgia among indigenous Australians (Ellis and Albrecht, 2017), and eco-grief resulting from diminishing sea ice and changing Arctic landscapes affecting the Inuit population (Cunsolo et al., 2013a; Petrasek et al., 2015; Cunsolo and Ellis, 2018). The link between suicide rates and crop-damaging temperatures causing economic hardship among Indian farmers has also been scrutinized (Carleton, 2017).

Empirical investigations focusing specifically on ecological emotions among vulnerable populations have predominately utilized qualitative methodologies (Tschakert et al., 2019; Middleton et al., 2020). The majority of this work emanated from indigenous communities in high-income countries like Canada, Australia, and the United States (Ojala et al., 2021). However, there was an increasing representation of populations from lower- and middle-income countries such as India (Carleton, 2017), Tuvalu (Gibson et al., 2020), South Africa (Chersich et al., 2018), and certain regions of China (Wang et al., 2020). These studies correlated extreme weather events and trends like temperature fluctuations, humidity, drought, and flooding to a range of psychological and behavioral outcomes, including PTSD, depression (Yang et al., 2011; Cheng et al., 2012), and even self-harming and suicidal tendencies (Qi et al., 2014; Burke et al., 2018).

For many indigenous natives and historically marginalized groups worldwide, the negative impacts of ecological emotions have run deeper by amplifying past-traumas of events such as forced displacement, systematically undermined traditional culture and continued colonization (Cunsolo et al., 2013a). These episodes of historical mistreatment and disempowerment often amplify feelings of helplessness and intense anxiety about future climates and livelihoods (Furberg et al., 2011; Cunsolo et al., 2013a). Specifically, American Indian and Alaska native communities routinely encounter a range of health inequalities, many of which are linked to their higher exposure to environmental health risks. Therefore, it is essential to incorporate their traditional practices and values into forthcoming strategies to minimize these environmental health hazards (Mayer et al., 2019). Indigenous peoples across the globe bear some of the most severe mental health repercussions of climate change (Middleton et al., 2020). The emotional toll taken by climate change can be acute among such populations, particularly when climatic shifts disrupt their customary practices and surrounding environment (Cunsolo et al., 2013b).

Geographical variations in eco-anxiety frequency have also been observed. To be frank, the majority of the studies cited herein are quantitative and predominantly conducted within developed Western contexts, but individuals from underprivileged areas are more prone to eco-anxiety, with women showing increased susceptibility to pre- and post-trauma stress (Patrick et al., 2023). Less affluent countries in the Global South have been found to express more concern about climate change’s impact on daily life than wealthier nations (Hickman et al., 2021), possibly due to differences in population susceptibility and adaptive capacities to climate change (Intergovernmental Panel on Climate Change, 2022). The latest multi-national study involving participants from China, India, Japan, and the U.S. also indicated that climate change anxiety was highest in Chinese and Indian populations (Tam et al., 2023).

With regard to mental health stressors, variations also appear based on gender and age. Exhibiting higher levels of ecological emotions, women, youngsters and older adults are particularly susceptible to climate-influenced mental health outcomes such as suicide and depression (Middleton et al., 2020). Female participants experienced higher levels of eco-anxiety (Searle and Gow, 2010; Berry and Peel, 2015; Verplanken et al., 2020). While men and women predict similar probabilities of severe impacts resulting from climate change, women worry more about potential outcomes (Sundblad et al., 2007). Compared to men, women exhibit more stress, anxiety, and behavioral engagement, even displaying higher rates of PTSD (Doherty and Clayton, 2011). Younger participants also express higher levels of eco-anxiety, with survey data suggesting an equal, if not higher, level of interest and concern regarding climate change among the younger generation than older individuals (Searle and Gow, 2010; Berry and Peel, 2015; Patrick et al., 2023). Being more likely to face the negative ecological impacts directly, young people seem to be more likely to accept the scientific consensus on human origins of climate change (Corner et al., 2015). Furthermore, high levels of concern about climate change have been observed among young people and children, with 84% of respondents indicating at least a moderate level of eco-anxiety (Hickman et al., 2021). Inuit youth in Canada express concerns about their future and the worsening environmental impact on their elderly natives (Petrasek et al., 2015). Interestingly, adolescents worrying persistently or increasingly about climate change were found to be more politically engaged than their moderately worried peers (Sciberras and Fernando, 2021).

## Individual resilience as an intervention strategy

In light of the escalating frequency and severity of climate change-related natural disasters over the past two decades, the emotional and psychological well-being of those individuals exposed directly or indirectly to the disasters has been significantly impacted. Generally speaking, resilience appears to be more common than pathological outcomes in these circumstances (Chen et al., 2020).

### Individual resilience as a positive intervention strategy

Although ecological emotions like eco-anxiety, eco-worry, eco-grief and eco-anger have negative impacts on individuals’ mental health, it remains unclear whether these negative emotions are necessarily maladaptive or pathological (Usher et al., 2019). In contrast, some researchers argued that these emotional states could exert a positive influence on implementing eco-friendly actions (Verplanken and Roy, 2013; Clayton and Karazsia, 2020) with implications for pro-environmental behavior (Verplanken et al., 2020), and that pro-environmental behaviors like climate activism could offset the impact of cognitive-emotional impairment from eco-anxiety on depressive disorder symptoms (Schwartz et al., 2022). Moreover, eco-anxiety has an adaptive aspect linked with expectations, motivation, and hope. According to Sangervo et al. (2022), eco-anxiety and hope could be seen as intertwined, potentially motivating humanity to find solutions for climate change. Individuals with higher levels of eco-anxiety at a given time were found to be more likely to engage in pro-environmental behaviors (Pavani et al., 2023).

To be specific, the concept of eco-anxiety has been noted to harbor positive implications, encompassing the potential for encouraging ecologically sustainable behaviors (Pihkala, 2020a,b). Problem-solving responses represent mindful efforts to modify or alleviate the impacts of distressing events, which are often considered a suite of actions aimed at resolving the problems that induce unpleasant emotions (Lazarus and Folkman, 1984). Therefore, being involved in climate advocacy might kindle a sense of achievement or satisfaction, suggesting a healthy way to mitigate the toll that eco-anxiety exerts on individuals’ mental health (Schwartz et al., 2022).

Some studies even suggested that non-clinical eco-anxiety should no longer be viewed as a mental and psychological disorder as it might drive people to adopt problem-solving-centered adaptive coping strategies, enabling eco-anxiety to play a constructive role in alleviating negative emotions through relevant behaviors (Clayton, 2020; Verplanken et al., 2020; Ojala et al., 2021). For instance, some researchers argued that an increase in eco-anxiety could positively affect environmental behavior intentions (Chu and Yang, 2019; Wullenkord et al., 2021) and make individuals more willing to participate in implementing relevant policies (Sciberras and Fernando, 2021). Dividing environmental behavior into promotive and compensatory types, Gao et al. (2021) once discussed the impact of eco-anxiety in conjunction with self-differences and concluded that with high self-differences, eco-anxiety increases compensatory pro-environmental behavior by stimulating feelings of guilt. From an existential perspective, Budziszewska and Jonsson (2021) made a further conclusion that eco-anxiety might make individuals pay more attention to the meaning of their existence and see environmental action as a means of obtaining significance.

Then it is reported that eco-anxiety is higher among younger people with higher ecological concern and greater nature-relatedness, but this anxiety might predict a motivating force for effective pro-environmental action (Whitmarsh et al., 2022). Back in 2007, Hicks and Holden (2007) reported that young people with negative views on the global future also expressed strong beliefs that they could influence the climate problem and make changes for a better future. In 2009, Taber and Taylor (2009) showed that teaching about climate change among middle-school children could increase both their concern about this problem and their sense that the problem could be prevented.

To a great extent, eco-worry also goes hand in hand with a feeling of personal influence over ecological problems. Consistent studies from various age groups and periods have shown that eco-worry positively affects pro-environmental behaviors and intentions (Hine and Gifford, 1991; Ojala, 2008; Sundblad et al., 2014; Hornsey and Fielding, 2016; Coelho et al., 2017; Ogunbode et al., 2019; Bouman et al., 2020). Some findings also tied eco-worry with support for environmental policy (Smith and Leiserowitz, 2014; Bouman et al., 2020; Goldberg et al., 2021). In fact, early in 1999, Hokka et al. (1999) found that teens from Finland greatly concerned about environmental issues felt more responsible for the environment and acted more eco-friendly. Then, Ojala’s (2007) study also found that individuals’ concern over climate change drives positive engagement in environmental protection. Moreover, Verplanken et al. (2020) discovered a notable correlation between habitual eco-worry and a pro-ecological worldview, and this relationship might extend to a green self-identity, pro-environmental conduct, and a personality characterized by openness, strongly hinting at a constructive nature.

Being primarily studied from a clinical perspective, eco-anxiety and eco-worry have been most often associated with negative effects such as low well-being and anxiety disorders (Sibrava and Borkovec, 2006; Barlow et al., 2019). However, an early study about the subjective meaning of nonclinical worry has shown that eco-worry might act as an emotional motivator to keep an individual alert and prepared for action and analytical thinking (Tallis et al., 1994). This aligned with some applied studies in political psychology, showing that anxiety and worry are precursors for deliberation and critical thinking (Valentino et al., 2008; Marcus et al., 2011). In this way, eco-anxiety and eco-worry might stimulate engagement with larger societal issues through critical thinking processes (Ojala et al., 2021). Yet, it was believed that these nonclinical eco-anxiety and eco-worry facilitated adaptive behaviors only when situations were perceived as controllable; when uncontrollable, they could instead culminate in stress and low well-being (MacGregor, 1991; Tallis et al., 1994). Furthermore, through an existential perspective, eco-anxiety and eco-worry were perceived as rational and frequently constructive responses to threats against individuals’ fundamental values, signifying a mature way of confronting human beings’ responsibilities (Ojala, 2016; Pihkala, 2020b).

Besides eco-anxiety and eco-worry, eco-grief and eco-anger were also considered not just as negative emotions but also as part of a coping process during which a person attempted to deal with the loss of vital relationships with the ecological environment (Lazarus, 1991; Kofod and Brinkmann, 2017). Eco-grief might be a part of an adaptive process though it sometimes leads to negative outcomes including persistent complex bereavement disorder and depression under harsh circumstances (Lazarus, 1991; Gross, 2016; Lenferink et al., 2019). Then, Kleres and Wettergren (2017) once reported that both hope and guilt might spark action-oriented anger which could transform fear into action. Eco-anger was even found to predict greater engagement in collective action than eco-anxiety and eco-grief (Stanley et al., 2021).

Furthermore, several studies have found moderately large positive correlations between climate change concern and efficacy beliefs among adults (Heath and Gifford, 2006; Kellstedt et al., 2008; Milfont, 2012), implying that people who feel threatened by climate change also feel more efficacious about their ability to tackle the issue. According to Hornsey et al. (2015), there might be a causal relationship between the perception of climate change threats and beliefs in efficacy. They proposed that elevated efficacy convictions could result from perceived threats as a part of a motivated coping attempt whereby acknowledgment of the threat posed by climate change also operated as a motive to believe that the threat might be mitigated.

## Limitation of individual resilience

To some extent, pro-environmental behaviors in the form of climate activism could soften the blow of climate change cognitive-emotional impairment on major depressive disorder symptoms caused by ecological emotions. However, such pro-environmental involvement might not effectively combat the negative impacts of ecological emotions as individuals might feel that their efforts make little difference in mitigating ecological crises (Boluda-Verdu et al., 2022), and then the ensuing frustration and despair could escalate due to such feeling. Just as Hickman et al. (2021) once argued, the root cause of global warming is primarily an unsustainable “system” (referring to politicians and corporations) and personal actions might not bring about significant progress. Moreover, among those who were equally concerned about climate change, the individuals feeling betrayed by those weak and lagging governmental responses were faced with increasing negative emotions.

The American Psychological Association characterized ecological emotions as a facet of personal mental health and well-being, and resilience was primarily regarded as an effective way for an individual to mitigate negative emotions and prevent declines in mental health and well-being (Clayton et al., 2017). This egocentric perspective is pervasive in the media and ingrained in the everyday language used to discuss the emotional impacts of environmental issues. Consequently, individuals grappling with ecological concerns and experiencing ecological emotions are often prompted to bolster their resilience to adjust personally to the emotional burden by governmental institutes, media and professional experts as they intend to guide different forms of psychological education and intervention. For instance, although eco-anxiety was described as an enduring fear of environmental catastrophe (Clayton et al., 2017), in many educational and media narratives, it’s not fear that matters, but rather a sense of personal responsibility accompanied by feelings of guilt and shame, which may be interpreted as a response to the internal conflict between a sense of personal responsibility, the urgency of climate change, and the enormity of the task which is impossible to achieve by any individual.

Indeed, ecological emotions have a significant social and political dimension. While the intent of boosting individual resilience aims to help individuals manage the threat of climate change, it often fails to account for the social factors behind the threat. Marks et al. (2021) once surveyed eco-anxiety across ten different countries, revealing that young people do not just worry about their future, but often feel betrayed by the inaction of political leaders. The feelings of betrayal and then helplessness reflect a lack of social environment support for sustainable lifestyles, pushing climate action onto individuals. Especially in the Global North, as there is a pronounced emphasis on individualism which holds individuals accountable for their life outcomes, the so-called logical approach to addressing ecological emotions focuses on resilience, that is, fortifying the coping capabilities of affected individuals so they can adapt to stress and persevere with their life tasks (Kałwak and Weihgold, 2022). As a result, inaction from government bodies, corporate sectors and massive industries related to ecological crises shifted the burden onto individual citizens. However, an individual is not equipped to address a global issue that affects all of humanity. In this way, individuals’ feelings of failure, depression and anxiety are amplified as they feel confronting severe ecological crises alone. Furthermore, the expense and burden of adaptation are often placed in local locations with fewer resources and power to avoid a reduction in national or international economic growth (Bottrell, 2009; Cannon and Müller-Mahn, 2010; MacKinnon and Derickson, 2013; McEvoy et al., 2013; Chapman et al., 2018).

The emotional impact of ecological problems is further deepened by a sense of abandonment by political and economic leaders, leading to feelings of helplessness and isolation (Weintrobe, 2012; Pihkala, 2020a). Although the promotion of collective action against ecological issues may mitigate this feeling of individual isolation, the sense of responsibility is construed as something of value and a driving force behind activism in activist circles. The problem is that, when activists frequently experience burnout and depression, the onus of coping with the emotional toll of combating ecological crises is often placed upon those individuals to preserve the physical and emotional resources necessary to sustain activism. Consequently, this discourse interweaves individual psychological aspects of ecological emotions with ethical and moral spheres, deeply affecting the emotional well-being of those individuals committed to ecological action.

In a word, recognizing ecological emotions requires attentiveness to their social and cultural elements as well as ethical considerations, particularly when implementing different types of psychological intervention. The current emphasis on individual resilience has been challenged due to its lack of attention to the root triggers of ecological emotions. To a large extent, this oversight may be attributed to the current research focus on the individual while treating the natural and social environment as secondary, and so this individualistic approach to ecological emotions perpetuates the transfer of responsibility for ecological crises to individuals. In the next part, we advocate for a multi-faceted counter-narrative that acknowledges sources of injustice and embraces diverse values.

## Collective engagement as a potential intervention strategy

Environmental psychology seeks to address a growing need for mental health interventions pertaining to the psychological effects of environmental crises. An important issue is to prevent established knowledge and interventions in mental health from validating and perpetuating climate injustice. To this end, indigenous cultures could offer some solutions to this conceptual impasse through counter-narratives that inspire innovative ways of thinking and creating interventions (Wilson, 2008; Kimmerer, 2013; Cutter-Mackenzie-Knowles et al., 2020; Kałwak and Weihgold, 2022). Hence, there should be a re-evaluation of psychological discourse which has idealized resilient individuals adapting to ecological crises, and this reorientation could foster a counter-narrative of interconnectedness, promoting a more sustainable, equitable society (Kałwak and Weihgold, 2022).

As eco-anxiety is often interpreted as an internal, psychological and emotional issue within individuals, the common response is to advocate for emotional resilience as the solution. However, this approach risks overemphasizing the needs, identities, and emotions of those who contribute to ecological issues, potentially allowing them to revert to an anthropocentric view of the world. In contrast to focusing on individual resilience, some researchers highlighted ecological emotions as a key catalyst behind collective engagement. For instance, Ojala (2007) found that young Swedish adults involved in environmental and global justice groups have acknowledged eco-anxiety as a key component in their involvement, and another study (Kleres and Wettergren, 2017) recognized the interplay of fear and hope in encouraging and maintaining engagement toward environmental activism. Yet, this emotional mix was predominantly observed among activists from the Global North while those from the Global South expressed feelings of fear, guilt, and anger, suggesting cultural or structural variations might influence emotional engagement related to climate change and other environmental issues (Ojala et al., 2021). In this way, while individualism erodes social connections, breeds isolation, and inaccurately represents ecological realities (Gillespie, 2020), engaging with feelings associated with nature and developing relationships with others who share the same experiences can ameliorate the stress related to ecological emotions. Therefore building relationships with others who resonate with our emotions is vital for sustaining emotional well-being (Kałwak and Weihgold, 2022).

In fact, for some people, worry about global environmental problems takes the form of macro worry with moral and ethical implications, as the worry is not centered around themselves or their loved ones but focuses more distantly on elements like people residing in foreign countries, animals, nature, and future generations (Lee and Barnett, 2020). This form of macro worrying is most prevalent among people who highly value global justice, peace, equality, and the welfare of nature and animals (Bouman et al., 2020; Helm et al., 2021). Moreover, it’s important to note that political orientation also plays an important role, with politically left-leaning persons expressing higher levels of worry than right-leaning ones (McCright and Dunlap, 2011; Gregersen et al., 2020). Hence, the concern people express is not solely due to scientific facts but also involves subjective aspects like values and political orientation.

In a word, sharing experiences within a group cultivates a sense of interconnectedness and provides actionable strategies to combat the negative effects of ecological emotions. However, this sense of connection is not limited to solidarity and support among human beings. Unlike the dominant Western perception of nature as a commodity or resource, indigenous beliefs appreciate the Earth as a living entity filled with spirit. In the indigenous perception, the Earth constantly provides food, water and shelter, and so humans, as the recipients, have the responsibility in return to preserve enough resources so that all beings can continue to thrive (Kimmerer, 2013). This perspective fosters a reciprocal relationship between man and nature, involving specific duties and responsibilities. As long as these obligations are fulfilled, a balanced relationship between humans and nature can be maintained (McGregor et al., 2020). Furthermore, this balance leads to a holistic view. Ross (1989): Preface II once put forward his holistic view that “American Indians are *all* one people.” Inspired by Eastern Buddhism, Zen and Taoism, Ross (1989, p. 32) observed that the symbol of wholeness of the psyche is a circle with designs representing balance, not the original symbol of Christianity (a circle with an “X” within), which, as he mentioned in Dr. Jung’s words, represents man’s belief in the delusion that he is superior to nature, suggesting the modern man in the predominantly Christian society is out of balance. Anyway, when discussing relationships from the indigenous perspective, it’s not only about human-to-human interactions, but also about the broader scope of relations with the more-than-human, such as the land, cosmos, and abstract ideas. As Boyd et al. (2023) once argued, eco-anxiety can be taken as an emergent form of posthuman knowledge, predominantly characterized by vulnerability rather than affirmation. Hence the fostering of ethical relationality through meaningful encounters with multiple species presents potential for transmuting this vulnerability and alleviating the anxiety.

To a great extent, reflecting on the contemporary world’s ecological crisis and the ensuing ecological emotions from the perspective of humanistic values has significant enlightening implications. Similar to Indians’ holistic view, classical Chinese philosophy features characteristics of organic wholeness. The concept of “Tian Ren He Yi” (天人合一) represents the “main keynote” of ancient Chinese philosophy (Liang, 2010: 59), and its basic meaning is “the intrinsic unity between humans and nature” (Meng, 2004: 5). Confucianism, Taoism, and Mohism all share the same kind of pursuit of the unity of humans and nature (Fang, 2013). Unlike Western culture, which tends to set humans and nature in opposition, the philosophy of “Tian Ren He Yi” advocates that humans should adapt to rather than conquer nature, which is a valuable ideological resource for alleviating ecological emotions caused by ecological crises (Fei, 2004). In this way, the idea of “Tian Ren He Yi” contains a wealth of ecological wisdom. Then, Confucianism advocates “Min Bao Wu Yu” (民胞物与), emphasizing the fraternal relationship between man, nature and all other things and asserting that humans should not dominate over nature. This humanistic concern of “Min Bao Wu Yu” exemplifies the practical implementation of the philosophy of “Tian Ren He Yi,” affirming the connection between all things in nature and humanity. It both acknowledges humanity’s use of nature for survival and development and restrains the exploitation of natural resources, embodying an aspiration for harmonious coexistence between humans and nature (Zhang, 2023). At last, Taoism promotes the principle of “Dao Fa Zi Ran” (道法自然), asserting that harmony between humans and nature, as well as among humans themselves, can only be achieved by adhering to and complying with the laws of nature.

Indeed, as a fundamental concept in traditional Chinese culture, humans and nature form a dynamic equilibrium in an organic system and humans have always coexisted with nature and all its creatures. To a great extent, Chinese traditional philosophy is a kind of Nature Philosophy, that is, the philosophy that observes, experiences, and interprets the universe, society and life from the perspective of nature (Lu, 2012). To Chinese people, the best state of life is to unite with nature to find peace of mind: they will try their utmost to forget the self and the object to be in harmony with nature (He, 1988). In fact, many Western ecological philosophers have found inspiration in Chinese philosophy. Nash’s ecological holism was deeply influenced by Chinese Taoism. To him, at the core of Taoism was “a rejection of the dualism and anthropocentrism that so thoroughly colored traditional Christianity”; by “advocating the submersion of the human self in a larger organic whole they cleared the intellectual way for environmental ethics” (Nash, 1989, pp. 112–113). Marshall also thinks that Taoists take a holistic view of the universe, recognizing the ecological principle of unity in diversity, as the whole is greater than the sum of its parts, in nature as well as in society” Marshall (1992).

## Discussion

Seen from the established literature, ecological emotions first attracted the attention of clinical psychology and then extended to fields such as environmental psychology. Based on the concept of trait anxiety and state anxiety, we first sort out the existing study of the concept of ecological emotions such as eco-anxiety, eco-worry and eco-anger, dividing clinical and non-clinical ecological emotions with the latter as our focus. Then, we summarize the research progress on ecological emotions, classifying the causes of ecological emotions from internal and external perspectives, and sorting out ecological emotions’ effects especially negative impacts on sensitive populations. At last, we examine the spectrum of ecological emotions and their multifaceted impacts on mental health, exploring intervention strategies from individual resilience to collective engagement.

We identify both individual resilience and collective engagement as crucial intervention strategies, yet their roles are not entirely complementary. Individual resilience tends to focus on psychological adaptation to stressors, often promoting personal coping strategies that may inadvertently support status quo attitudes toward environmental degradation. This inward focus, while beneficial for personal mental health, can be critiqued for not addressing the broader socio-political frameworks that perpetuate ecological crises. On the other hand, collective engagement is framed as a more inclusive and potentially transformative approach. It not only addresses the emotional toll on individuals but also fosters a shared sense of purpose and action that could lead to systemic change. This strategy is particularly poignant in its inclusion of the holistic perspectives of indigenous and Chinese philosophy, which offer a relational understanding of nature and emphasize collective over individual well-being.

We capture the influence of socio-cultural contexts on the experience and expression of ecological emotions. Vulnerable populations, including indigenous peoples and residents of developing countries, often experience more intense ecological emotions due to direct and profound connections with their environment. The interplay of cultural values, media influence, and political ideologies also shapes how ecological emotions are perceived and acted upon, suggesting that any intervention must be culturally sensitive and aware of these dynamics to be effective.

Indeed, it is out of these ecological emotions’ significant repercussions on vulnerable communities that we endeavor not only to underscore the plights of individuals but also to reimagine potential pathways through which these emotional responses can be transformed from private distress to collective empowerment. Central to this dialog are the elements of indigenous wisdom and the foundational concepts of traditional Chinese philosophy, which when interwoven offer a richer, more communal paradigm for ecological activism.

Indigenous cultures across the globe have long held a profound connection with the Earth, embodying a symbiotic relationship that regards nature not as a resource to be exploited but as a living entity to be respected. This relational understanding fosters a collective conscience and acknowledges the intrinsic value of all life forms. Indigenous teachings such as those of the Indian concept of “Mitakuye Oyasin” (We are all related) invite us to see beyond the confines of individualism and recognize our shared responsibility toward the Earth and each other. Similarly, traditional Chinese philosophy imbues its connectedness with nature through principles like “Tian Ren He Yi.” These ideologies promote harmony between humans and the natural world, guiding us to model societal structures and personal behavior on the patterns and cycles inherent in nature.

By drawing on these ancient wellsprings of knowledge, we can anchor ecological activism not in the pathology of distress, but in the recognition of a shared destiny and mutual belonging. The sense of urgency stimulated by ecological emotions can thus be rechanneled from isolating experiences of anxiety, stress, or depression into a potent force for community-driven change. We suggest that ecological emotions, when collectively acknowledged and directed, have the potential to galvanize a form of activism that is deeply rooted, sustainable, and imbued with care for the community and our environment. To handle the growing ecological emotions efficiently, we had better jump out of the limitation of human-to-human interactions of Western anthropocentrism to pursue the unity of humans and nature with the help of the holistic and collective wisdom of indigenous cultures and Chinese traditional philosophy.

In the interpretation of the findings, our results have to be treated with caution since the methodological foundation of the research was less than ideal. Most studies were conducted cross-sectionally and the pool of data for the narrative review was somewhat limited. A more focused examination of this global health concern is certainly warranted as we move forward, and so a big challenge lies in evaluating each ecological emotion independently. Yet, a more complicated priority is how to make full use of the holistic wisdom of indigenous cultures and Chinese traditional philosophy to explore the potential change of nonclinical intervention strategy from Western individual resilience to the holistic and collective engagement.

## Author contributions

SQ: Writing – review & editing, Conceptualization, Writing – original draft. JQ: Writing – review & editing, Conceptualization, Writing – original draft.
